# Clinical insights into the three-dimensional anatomy of cheek teeth in alpacas based on micro-computed tomography. Part 1: mandibular cheek teeth

**DOI:** 10.1186/s12917-021-03038-x

**Published:** 2021-10-22

**Authors:** Kirsten Proost, Matthieu N. Boone, Ivàn Josipovic, Bart Pardon, Koen Chiers, Lieven Vlaminck

**Affiliations:** 1 Department of Surgery and Anesthesiology of Domestic Animals, Faculty of Veterinary Medicine, Ghent University, Merelbeke, Belgium; 2 Department of Physics and Astronomy – Radiation Physics, Faculty of Science, RP-UGCT, Ghent University, Ghent, Belgium; 3 Department of Large Animal Internal Medicine, Faculty of Veterinary Medicine, Ghent University, Merelbeke, Belgium; 4 Department of Pathology, Bacteriology and Poultry Diseases, Faculty of Veterinary Medicine, Ghent University, Merelbeke, Belgium

**Keywords:** Alpaca dentistry, Apical infection, Common pulp chamber, Dental anatomy, Dental disease, Dental pathology, New world camelids, Sub-occlusal dentinal thickness, Tooth root abscess

## Abstract

**Background:**

Despite dental disease being a common health concern in alpacas, important dental pathology including apical infection, remains poorly understood. Treatment options are limited compared to veterinary dentistry techniques in other species. The primary goal of this study was to increase understanding of the external and internal anatomy of mandibular cheek teeth to enable the development of tooth sparing techniques in this species. Also, an objective evaluation of the sub-occlusal dentinal thickness in normal mandibular cheek teeth is warranted to understand the risks associated with reduction of overgrown teeth.

**Results:**

Overall pulp anatomy was variably characterized by the presence of a common pulp chamber in younger teeth, and segmentation of pulp cavities into multiple separate pulp entities within the same tooth with increasing age. A common pulp chamber was identified in 55.3% (26/47) of teeth with a mean dental age of 1 year and 11 months (± 1 year and 8 months). Columnar segmentation was recorded in the remaining teeth with a mean dental age of 6 years and 5 months (± 3 years and 11 months). Age of segmentation of the common pulp chamber into multiple separate pulp entities shows wide variation and is dependent of the specific Triadan position. The present study illustrates the presence of disto-mesial root contacts between adjacent tooth roots, often leading to morphological adaptations, most frequently observed between Triadan 09–10s (80%) and 10–11s (67%). The measured sub-occlusal dentinal thickness was as low as 1.11 mm over some pulp horns. The sub-occlusal dentinal thickness was lower than 2, 3, and 4 mm in 13.1, 38.1 and 61.4% of performed measurements, respectively.

**Conclusion:**

This study provides detailed information on age-dependent mandibular cheek teeth anatomy in alpacas, which may support the use and development of advanced dental treatments in this species such as endodontics and tooth sectioning techniques. Apical morphological adaptations caused by disto-mesial root contact between adjacent mandibular cheek teeth are clearly illustrated. The limited amount of sub-occlusal secondary dentin warrants a cautious approach with regards to dental floating in alpacas.

## Background

Dental disease is consistently described as a major health concern in alpacas. Tooth root abscesses, also termed apical infections, have received great attention in specific veterinary literature [1–3]. Recent studies have attributed to novel insights into the prevalence of specific dental disorders in alpacas [4, 5]. Several wear abnormalities at the level of the cheek teeth, including step mouth, wave mouth, enamel overgrowths, focal overgrowths and accentuated transverse ridges have been proven to be of importance in this species [4]. Floating of cheek teeth in animals suffering from these specific conditions can be beneficial for the prevention of more severe disease processes and/or the improvement of oral comfort, thereby ameliorating animal welfare. Despite the wide variety of available techniques for the treatment of dental disease in other species, options in alpacas remain relatively restricted and often consist of exodontia [6, 7]. More advanced dental treatments comprising endodontics are performed only in rare cases given the lack of in-depth anatomical knowledge of cheek teeth in this species. As known from other species, detailed knowledge of the anatomy of the pulp system is essential for the performance of successful endodontic treatments [8–10]. Further development of these modern ‘tooth saving’ techniques can reduce the need for exodontia in this species. Also when correcting wear abnormalities, knowledge regarding the distance between the occlusal surface and the pulp cavity is crucial to avoid iatrogenic pulp exposure and prevent thermal pulp damage.

To date, general knowledge regarding cheek tooth anatomy in this species is limited [11]. Historically, it was stated that mesial and distal pulp horns of cheek teeth in alpacas do not communicate [3, 12]. Recent research using computed tomography (CT) has demonstrated the presence of common pulp chambers in younger cheek teeth in New World Camelids [11]. Similar findings have also been reported in other hypsodont species [8, 13]. With increasing dental age, secondary dentin is believed to be laid down over the pulp cavity walls causing separation of the common pulp chamber (CPC) and creating separate mesial and distal pulp compartments [8, 11]. However, the precise moment at which separation of the CPC takes place in cheek teeth of alpacas is not known. Also, specific scientific data on the sub-occlusal dentinal thickness (SODT) at the level of the mandibular cheek teeth is lacking in alpacas.

The objective of this study was to describe the normal (pulpal) mandibular cheek tooth anatomy in alpacas and to describe age-related changes. Additionally, the SODT occlusal to specific pulp horns in animals of varying age is further investigated.

## Methods

### Specimen and study design

A cross-sectional study design was used. Fourteen hemimandibles were removed from the cadaver heads of 13 Huacaya alpacas, using an oscillating saw. Owner consent was obtained before the cadavers were released for the study. All animals died or were euthanized for non-dental reasons. No animals were euthanized for the purpose of the study. All cheek teeth were grossly examined for the presence of dental disease. Only cheek teeth in which the results of the required measurements could not be biased by a concurrent disease process were included in the dataset. Abnormalities which led to exclusion included accentuated transverse ridges, severe periodontal disease, occlusal pulp exposure and a defect in the apical region of the infundibular enamel leading to excessive production of reactive dentin. The latter was only visible when processing the results of the images acquired via micro-computed tomography (μ-CT), hence led to exclusion at a later stage. In total, 47 cheek teeth were retained from 13 animals. The sample pool consisted of 12 deciduous premolar, 8 permanent premolar and 27 molar cheek teeth. The sample size was primarily determined by availability of suitable samples and finances. The power of this sample size would allow detection of a difference of 1.5 mm in mean SODT between two dentition types, if an estimated standard deviation of 1.0 mm was taken into account and if 7 teeth per test group were available (mean SODT permanent premolars 5 mm – mean SODT molars 3.5 mmm). The study included 7 female, 4 male and 2 male castrated alpacas. Age of the animals ranged from 6 months to 15 years and 6 months with a mean of 5 years and 4 months ± SD 4 years and 7 months. Individual teeth were identified using the modified Triadan system [14]. The dental age of distinct cheek teeth was calculated to take the staggered eruption times of the different cheek teeth into account [15]. A description of the specific dental ages of included teeth according to Triadan position can be found in Table 1.

**Table 1:** Age distribution of the examined mandibular cheek teeth

				DA range		
	Triadan	# Teeth	Mean standard eruption age	Youngest	Oldest	Mean DA ± sd
Deciduous	07	6	0d	5m22d	3y	1y9m ± 1y
	08	6	0d	5m22d	3y	1y9m ± 1y
Permanent	08	8	4y2m15d	15d	11y3m15d	4y2m ± 3y8m
	09	10	7 m15d	0d	10y4m15d	3y10m ± 3y10m
	10	9	1y8m15d	7 m	13y9m15d	5y9m ± 4y9m
	11	8	3y2m15d	1y0m15d	12y3m15d	5y2m ± 3y8m

The mean standard eruption age is calculated based on the reported range of eruption times of each specific tooth [15]. The dental age (DA) is calculated based on the age of the animal and the mean standard eruption age. Dental age is recorded in years (y), months (m) and days (d)

Well-defined measurements were performed on a selected subset of teeth, after considering the dental age and health status of each cheek tooth.

### Scanning parameters

High resolution μ-CT scanning of complete mandibular arcades was performed in the custom-built scanner system HECTOR at the Ghent University Center for X-ray Tomography [16]. For each sample, 2001 projection images with an exposure time of 1 s per projection were acquired over an angular range of 360°. Using geometrical magnification, an isotropic voxel size in the reconstruction of 55^3^ μm^3^ was achieved. The tube was set to a high voltage of 180 kV and a target (output) power of 40 W, resulting in a negligible influence on the image resolution. To reduce beam hardening effects, a filter of 3 mm Aluminium was used. The projection data was reconstructed into a 3D volume using the implementation of the FDK algorithm in the in-house developed package Octopus Reconstruction. A commercial 3D-rendering software package (VGStudioMAX and myVGL, Volume Graphics GmbH,Germany) was used to generate 3D renderings and orthogonal slicing of the mandibular arcade and pulp system of included teeth and to perform specific measurements for each included cheek tooth.

### Constructing reference planes

**Fig. 1:**
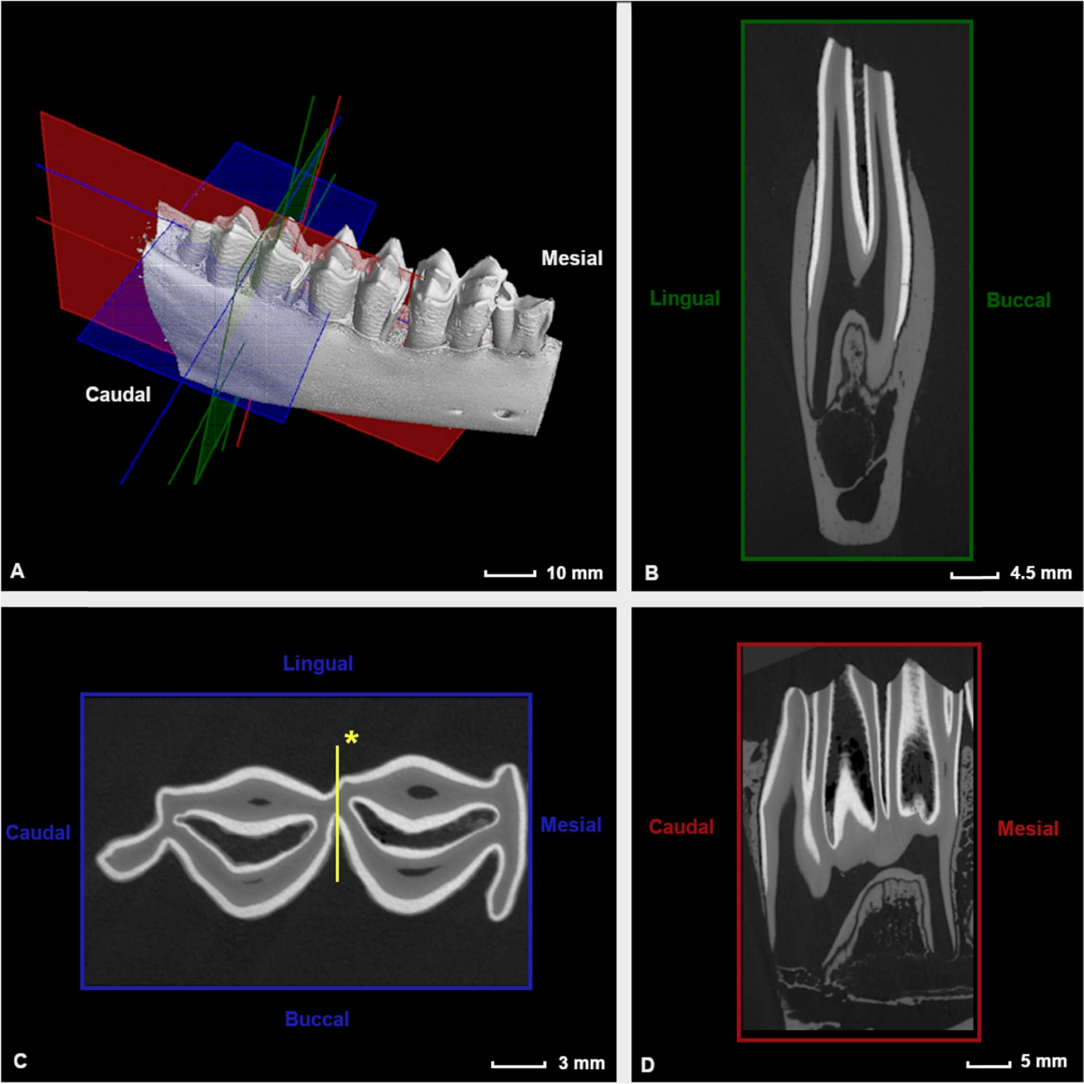
Reference planes constructed for the interpretation and comparison of anatomical aspects of alpaca cheek teeth as illustrated in a Triadan 411 of a 4 years and 3 months old alpaca. Subdivision between the mesial and middle column is illustrated on plane C (*)

To allow for standardized measurements and descriptive characterization of dental structures, three reference planes were drawn for each individual columnar structure in every cheek tooth (Fig. 1).

The first (B, green) was drawn from the most apical enamel point of a specific column following the longitudinal axis of this column. The second (C, blue) was set perpendicular to the longitudinal axis of the studied column. A third reference plane (D, red) was oriented in a mesiodistal plane and aligned with the infundibula present in that specific tooth. A 90° angle was maintained between all constructed reference planes.

### Descriptive morphology and measurements

**Fig. 2:**
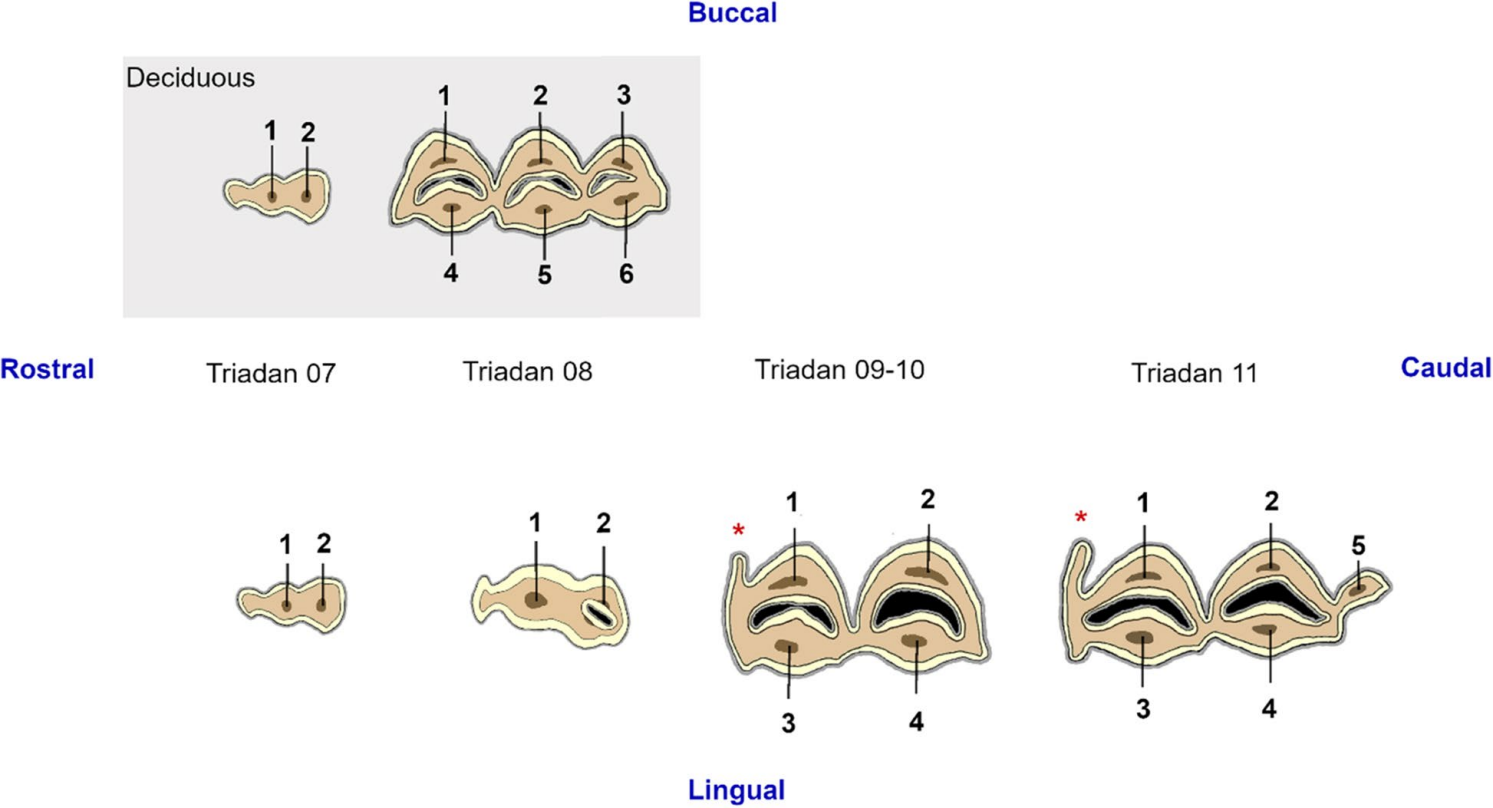
Pulp numbering system in alpaca mandibular cheek teeth adapted from the equid numbering system by du Toit et al. [18]. Deciduous teeth are displayed in a grey box. The occlusal surface consists of cement (dark grey), enamel (light yellow), dentin (light brown), darker secondary dentin overlying pulp horns (dark brown) and the lumina of the infundibula (black). Different pulp horns are numbered with Arabic numerals (1 to 6). The lama buttress is demonstrated on Triadan 09 to 11, marked by a red asterisk

The 2 and 3-dimensional general and pulpal anatomy of studied teeth was evaluated. A differentiation was made between crown and roots at the level of the bifurcation found in each tooth, with the roots located most apically. At crown level, different (pre) molars showed pronounced infolding of peripheral enamel which divided these teeth in 2 to 3 vertical columns (Fig. 1C, *). Infundibula were defined as funnel shaped invaginations or cups of enamel starting at the level of the occlusal surface [17]. Each column contained a maximum of one infundibulum. An endodontic numbering system was used based on the numbering system widely accepted in equids [18] (Fig. 2).

**Fig. 3:**
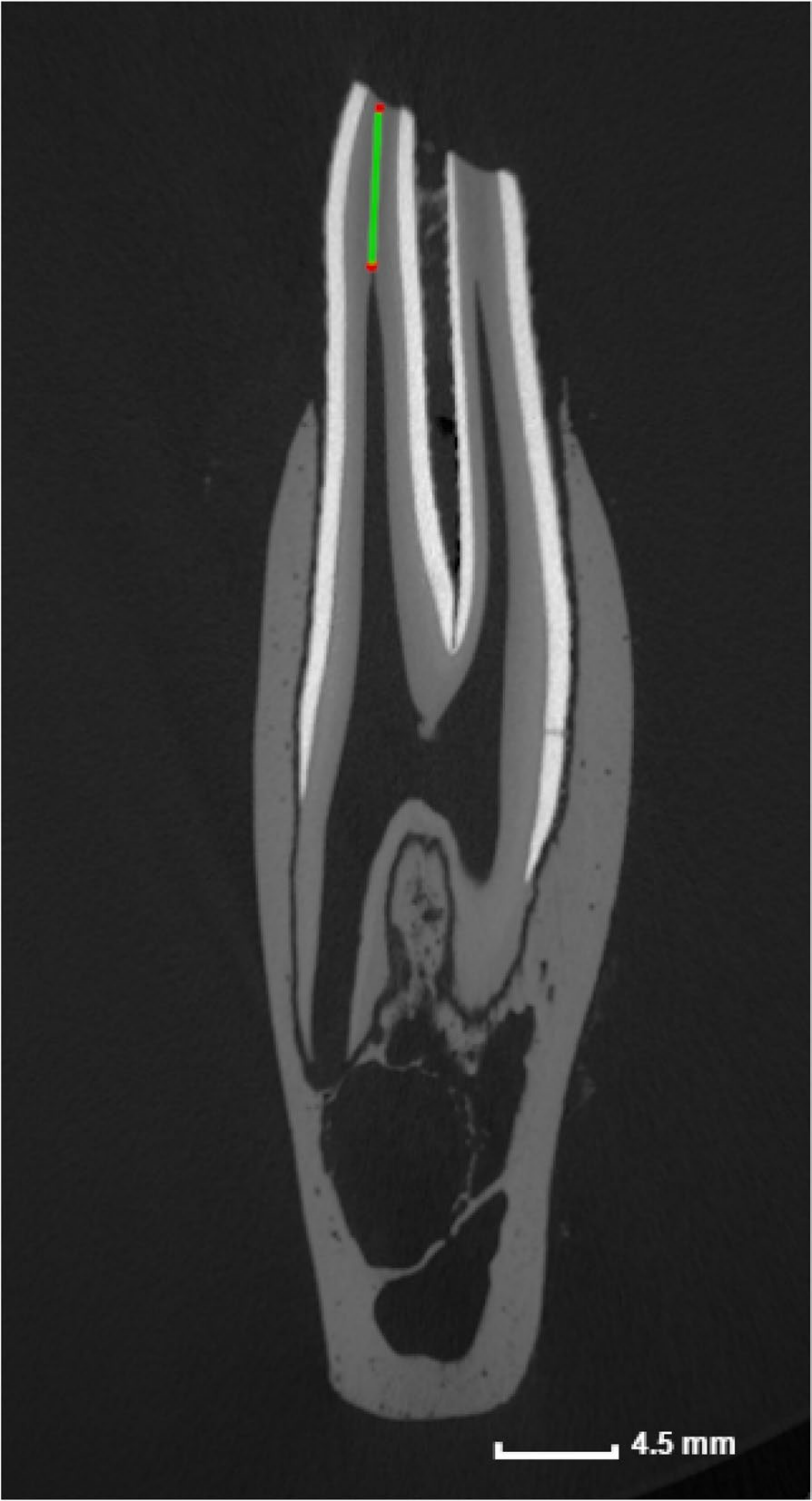
Standardized measurement of sub-occlusal dentinal thickness. Sagittal micro-CT section of a Triadan 411 of a 4 years and 3 months old alpaca illustrating standardized measurement of subocclusal dentinal thickness (in mm) overlying pulp horn 4 using reference plane B (Fig. 1). This measurement was performed for each individual pulp horn of all examined teeth

Strings of pulpal tissue extending occlusally from the pulpal coalescences were termed pulp horns, whereas apical extensions from the coalescences were termed root canals. A common pulp chamber (CPC) was defined as a pulp system in which communications exist between all identified root canals and pulp horns within the same tooth [8]. In contrast, pulpal segmentation was identified when isolated pulp compartments were found within the same tooth lacking communication with neighboring pulp compartments. This was defined as columnar segmentation (CS) if pulpal segmentation was specifically creating pulp segments within separate columns within the same tooth leading to mesial, (middle) and distal pulp compartments. Additionally, the SODT overlying individual pulp horns was measured as illustrated in Fig. 3.

### Statistical analysis

**Fig. 4:**
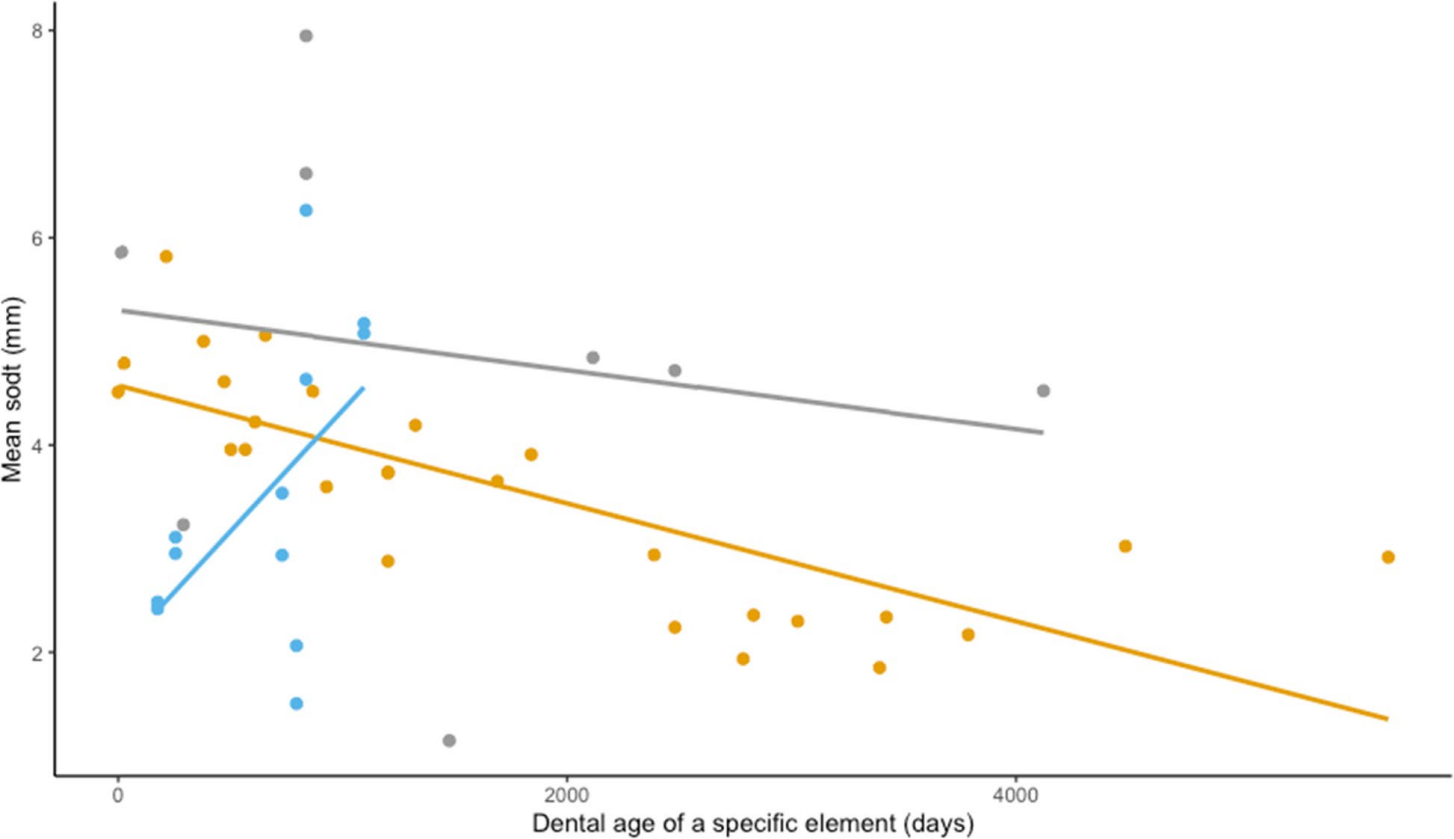
Scatterplot of the mean sub-occlusal dentinal thickness (SODT) of a specific tooth in function of the dental age (days). Values and fitted specific regression lines of the deciduous premolars, permanent premolars and molars are depicted in blue, grey and orange, respectively. A strong interaction between fixed effects ‘dental age’ and ‘type of dentition’ is evident. Mean SODT appears to increase with dental age in permanent premolars, whereas the opposite effect is noted in the other types of dentition

Descriptive statistics were performed on the degree of pulpal segmentation, dental age and sub-occlusal dentinal thicknesses of all included cheek teeth. Dental age (in days) was a first variable of interest. A first linear mixed model (lmer) was constructed with ‘Individual alpaca’ added as random factor. Mandibular arcade (left/right) and ‘Degree of pulp segmentation (CPC/CS)’ were tested univariably. Only ‘Degree of segmentation was significantly associated with dental age, unabling further model building. The mean SODT for all included teeth was calculated as a second variable of interest, given the strong differences in pulp morphology between the different types of dentition. To account for possible clustering, a random factor ‘alpaca’ was added to a linear mixed model for further statistical analysis. Several fixed effects including ‘Type of dentition (deciduous premolar/permanent premolar/molar)’, ‘Dental age (in days)’, ‘Gender (M/MC/V)’ and ‘Mandibular arcade (left/right)’ were tested univariably. A strong interaction seemed to exist between significantly associated factors ‘Type of dentition’ and ‘Dental age’, necessitating a split in the dataset to draw definite conclusions (Fig. 4).

A third linear mixed model was built with the mean SODT at the level of the mandibular molars as variable of interest. Given the strong structural similarities between the different molars, the specific SODT measured above a specific pulp horn of all included molar cheek teeth was additionally utilized as additional variable of interest. Univariable analyses with ‘Triadan position’ nested within ‘individual alpaca’ added as random factors, have been performed for aforementioned fixed effects. Furthermore, ‘Pulp horn’ was additionally tested univariably. Given the lack of multiple significantly associated fixed effects, no further model building was performed. Satterthwaite’s degrees of freedom method was used to obtain the *P*-values in each of the models. Normality was confirmed using a QQ-plot and the Shapiro-Wilk test on the residuals. To assess linearity, residuals were plotted versus fitted values. Residual plots were constructed to confirm the absence of homoscedasticity and potential outliers. Statistical significance was set at *P* < 0.05. Statistics were carried out using R V3.5.2., R Foundation for Statistical Analysis.

## Results

### General anatomy

The number of mandibular cheek teeth per mandibular arcade varied from 3 to 5. All cheek teeth had two roots but the morphological characteristics of their crowns differed remarkably between Triadan positions (Fig. 2). A small deciduous 07 and a larger deciduous 08 were found in the 6 youngest of studied mandibular arcades (mean age 1 year and 9 months ± 9 months). Both aformentioned teeth could not be found in animals older then 3 years. The deciduous 07 teeth were characterized by one column and no infundibulum. Whereas, the deciduous 08 showed a large ‘rectangular’ shape and consisted of 3 columns each with an infundibulum. The two mesial columns consistently joined apically to form the mesial root. The distal column continued to form the distal root. Remarkably, no permanent 07 was detected in our study population. A permanent 08 was seen in all animals older than 4 years and 3 months. This tooth solely consisted of 1 column with one infundibulum. Triadan 09s and 10s were grossly similar in shape, consisting of 2 columns each with an infundibulum. Triadan 11s were characterized by an additional distal column without the presence of an extra infundibulum. The 2 distal columns shared the same distal root. At the moment of tooth eruption, all mandibular molars were also characterized by the presence of a specific enamel fold at the level of the mesio-buccal border, known as ‘llama buttress’ [15]. This additional enamel fold was found at the occlusal surface in Triadan 09s and Triadan 10s up to 5 years and 11 months, and 13 years and 10 months of dental age, respectively. The llama buttress was found in all Triadan 11s in our study population.

### Number of pulp horns and pulp anatomy

**Fig. 5:**
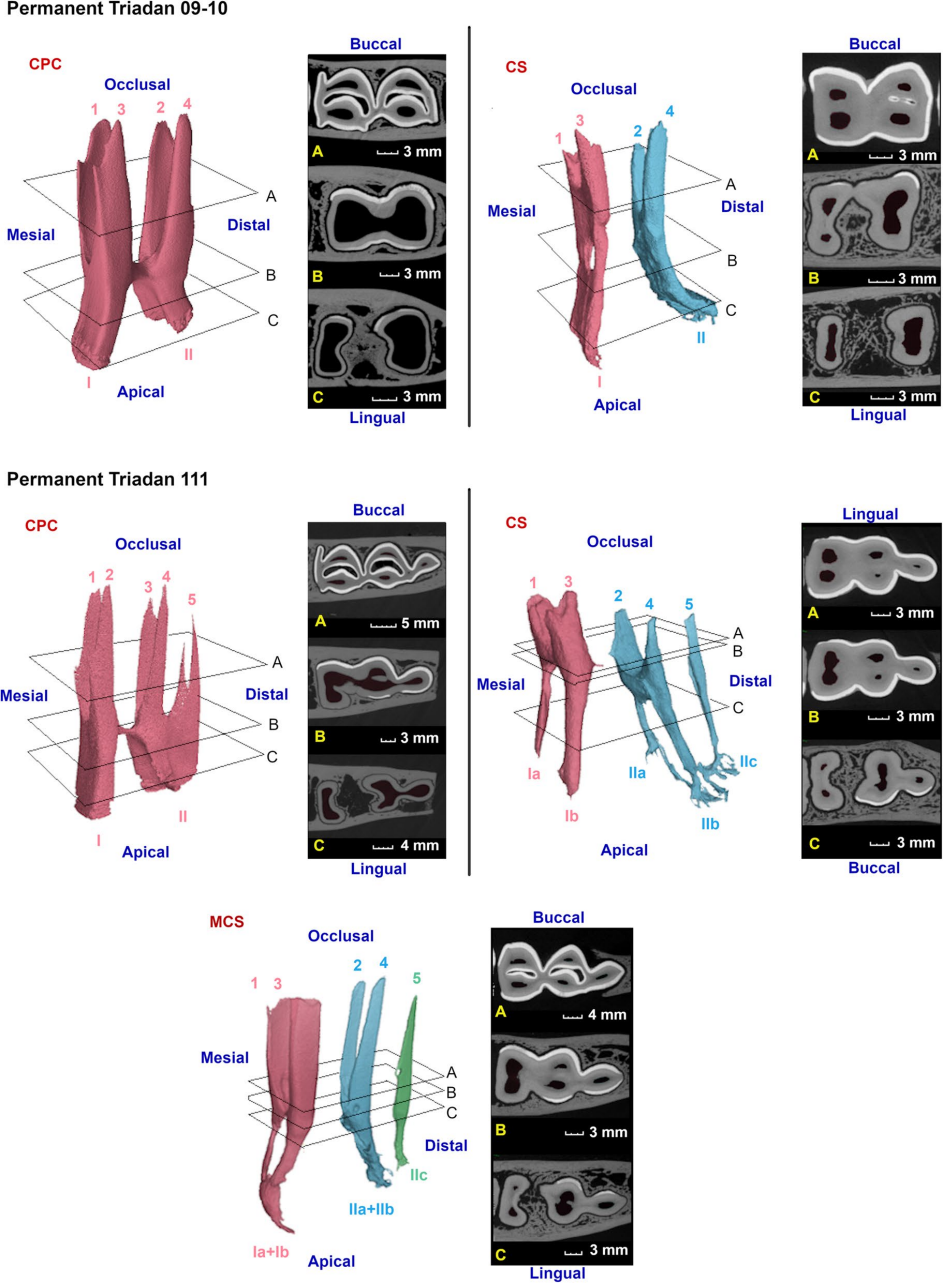
(See legend on next page). (See figure on previous page). Three-dimensional reconstructions of common pulp configurations in permanent mandibular cheek teeth in alpacas. Different colors have been used to indicate separate pulp compartments. Pulp horns and root canals are labeled with Arabic numerals ranging from 1 to 5 and Roman numerals ranging from I to IIc, respectively. Reference planes (A to C) indicate the position of selected 2D-images. Permanent Triadan 09s and 10s showed 2 different configurations being a CPC illustrated in a 1 years and 4 months old tooth, and column segmentation (CS) as illustrated in a 6 years and 7 months old tooth, creating two separate pulp compartments. In these teeth, root canal I is connected to pulp horns 1 and 3, whereas root canal II is connected to pulp horn 2 and 4. In permanent Triadan 11s, that have an additional pulp horn 5, 3 pulp configurations were recorded. A CPC is demonstrated in a tooth 1 year and 1 month post-eruption. Note that pulp horn 5 exceptionally splits in 2 more occlusally. CS is illustrated in a 13 years and 10 months old tooth. Root canals Ia (lingual) and Ib (buccal) are connected to pulp horns 1 and 3. Communication exists apically between root canals IIa, IIb, IIc and pulp horn 2,4 and 5. A third configuration was characterized by 3 separate pulp compartments in a 7 years and 10 months old tooth, creating maximal columnar segmentation (MCS). Note the splitting, followed by apical fusion at the level of root canal I which was specific to this tooth

**Fig. 6:**
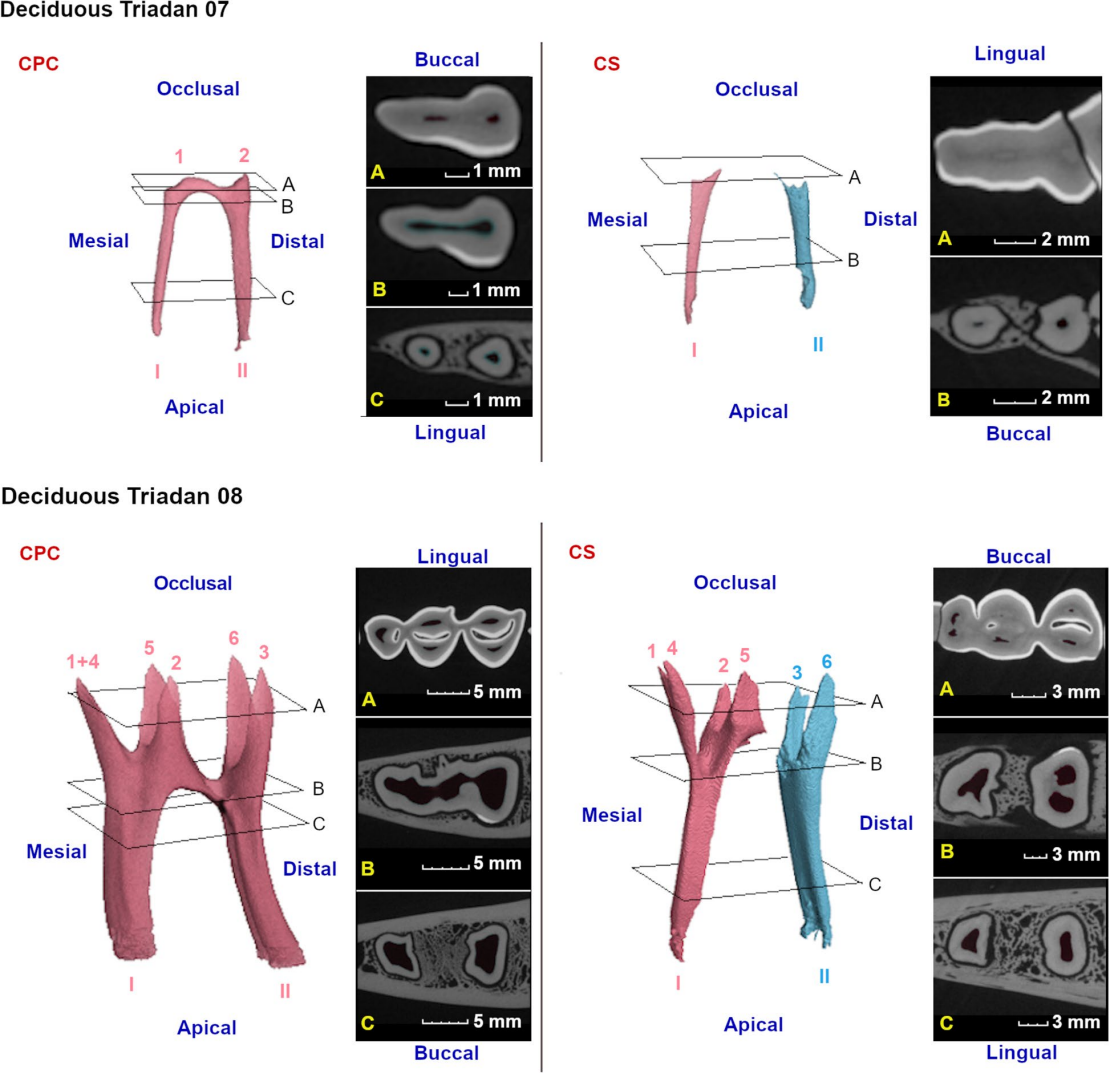
Three-dimensional reconstructions of observed pulp configurations at the level of the deciduous mandibular cheek teeth. Different colors have been used to indicate separate pulp compartments. Pulp horns and root canals are labeled as in Fig. 5. Reference planes (A to C) indicate the position of selected 2D-images. The deciduous Triadan 07 shows two configurations. In younger teeth, a wide communication exists between adjacent pulp horns and root canals as demonstrated in a 6 months old tooth (CPC, common pulp chamber, 807). With increasing age, secondary dentin is laid down causing narrowing or in this particular case, disappearance of the CPC in a 2 years and 4 months old alpaca. A deciduous Triadan 08 shows 5 pulp horns and a CPC in a young tooth of 6 months (808). In older teeth (708, 2 years) 6 pulp horns can be perceived. Columnar segmentation (CS) occurs with pulp horn 1, 4, 2 and 5, and root canal I remaining connected. The second pulp compartment in these teeth consists of pulp horn 3, 6 and root canal II

**Fig. 7:**
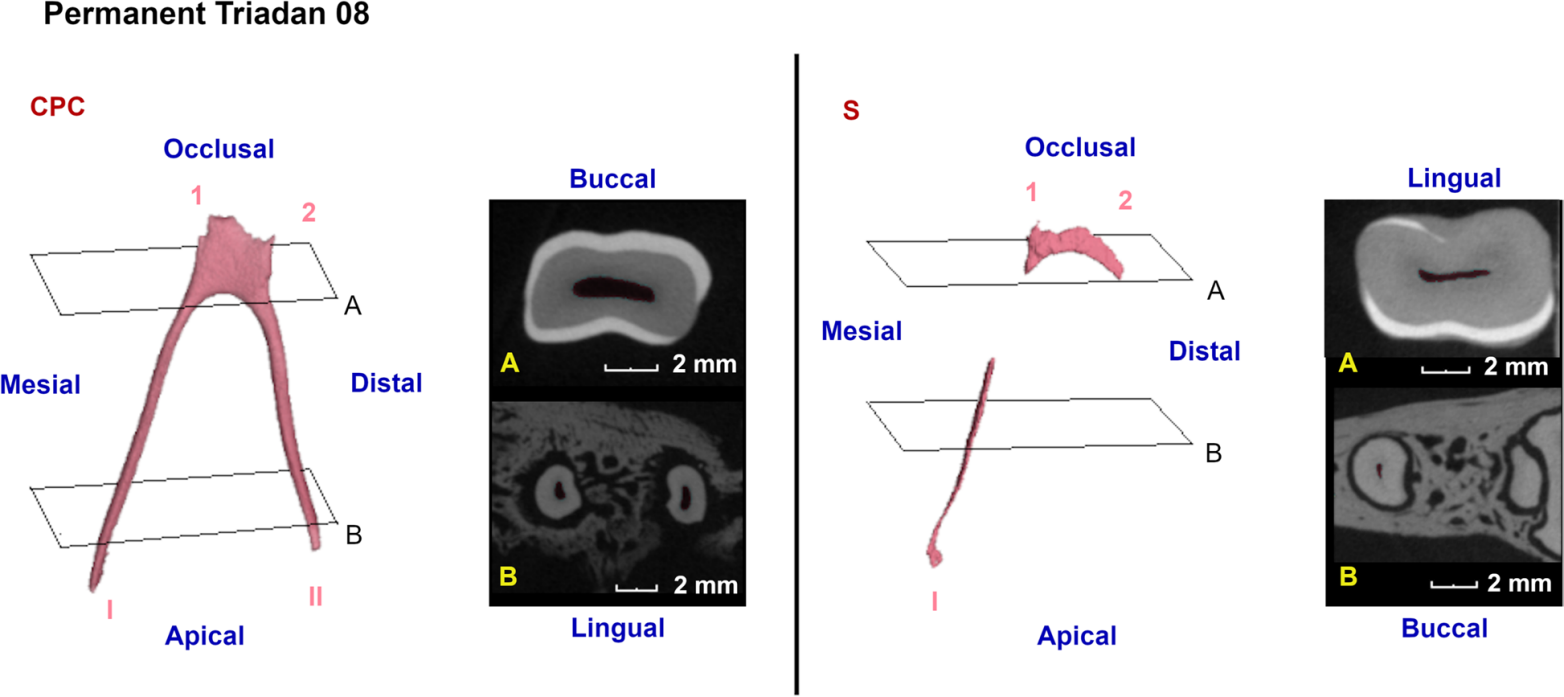
Three-dimensional reconstructions of the observed pulp configurations at the level of the permanent mandibular premolars. Pulp horns and root canals are labeled as in Fig. 5. Reference planes (A to C) indicate the position of selected 2D-images. A common pulp chamber (CPC) was observed in almost all teeth as demonstrated using a 3D-reconstruction of the pulp system of a Triadan 408 originating from a 5-years-old alpaca. Two minimal pulp horns can be identified. In a 15,5-years-old alpaca (S, dental age 11 years and 4 months), narrowing of the common pulp chamber was visible in an occlusal-apical direction. Communication between the mesial root canal and the central pulp pulpal coalescence was lost. Extensive production of secondary dentin in this tooth has led to a disappearance of root canal II

A large variation in pulpal anatomy was found between different mandibular cheek teeth (Figs. 5, 6 and 7). Deciduous Triadan 07 teeth showed 2 pulp horns and deciduous Triadan 08s contained 6 pulp horns, whereas only 2 pulp horns were identified in the permanent 08s. The central mandibular molars (Triadan 09–10) contained 4 pulp horns (Fig. 2). All studied mandibular Triadan 11s contained an additional pulp horn (5) at the distal edge of the tooth. The length of the roots varied in relation to dental age, with younger teeth having shorter roots with a wider apical foramen. Dental age also influenced the morphology of the root canal present within each root with a simple straight root canal identified especially in all freshly erupted teeth and a varying presence of apical deltas frequently observed in older cheek teeth (Fig. 5).

Overall pulp anatomy was variably characterized by the presence of a CPC in younger teeth, and segmentation of pulp into multiple separate pulp entities within the same tooth in older teeth. A CPC was identified in 26 of 47 (55.3%) mandibular cheek teeth with a mean dental age of 1 year and 11 months (± 1 year and 8 months). CS was recorded in 21 (44.7%) teeth with a mean dental age of 6 years and 5 months (±3 years and 11 months). Within a segmented pulp entity, communication persisted between all associated pulp horns and root canals. Mandibular cheek teeth with CS were significantly older then cheek teeth with a CPC (*P* < 0.001). Detailed characteristics of CPC differences in mandibular cheek teeth are shown in Table 2.

**Table 2:** The presence of a common pulp chamber (CPC) in examined mandibular cheek teeth

					# Pulp compartments		
	Triadan	% CPC	Youngest loss of CPC	Oldest CPC	1	2	3
Deciduous	07	66.7%	2y3m15d	2y2m3d	4	2	
	08	50.0%	2y	2y2m3d	3	3	0
Permanent	08	87.5%	11y3m15d	6y9m15d	7	1	
	09	50.0%	2y4m15d	1y8m	5	5	0
	10	44.4%	2y6m15d	4y7 m15d	4	5	0
	11	37.5%	3y3m15d	3y3m15d	3	4	1

The dental age of teeth in specific CPC categories is displayed in years (y), months (m) and days (d).

A CPC was found in 4 out of 6 (66.7%) examined deciduous 07s. Once exceeding the dental age of 2 years and 4 months, the pulp horns and central pulp region were completely obturated with secondary dentin and the root canals did not show any remaining communication (Fig. 6). Deciduous 08s showed a CPC until the age of 2 years and 3 months. In older teeth, segments consisting of pulp horns 1, 2, 4, and 5 (columns 1 and 2) fused with root canal I, and pulp horns 3 and 6 (distal column) fused with root canal II. In the oldest examined deciduous 08 (dental age of 3 years), root canal II split into a lingual and a buccal canal.

Almost all studied permanent 08s showed a CPC (7/8, 87.5%). In the oldest tooth (11 years and 4 months), communication between the central pulp region and root canal I was lost and root canal II was obliterated due to secondary dentin deposition (Fig. 7, S).

A CPC was recorded in 50 and 44.4% of Triadan 09s and 10s, respectively. Segmentation of the pulp system in the remaining teeth led to two separate pulp compartments which included pulp horns 1 and 3 and root canal I in the mesial, and pulp horns 2 and 4 and root canal II in the distal column (Fig. 5). CS was seen in the majority of the studied Triadan 11 s (5/8, 62.5%). In 3/5 of these (dental age < 6 years and 10 months), segments included pulp horns 1 and 3 and root canal I (a + b), and pulp horns 2, 4 and 5 and root canal II (a + b + c). In one specific Triadan 11 (dental age of 12 years and 4 months) the distal segment showed further columnar separation of pulp horn 5 that only communicated through an additional distal root canal with the root canal (root canal IIc) associated with pulp horns 2 and 4 (Fig. 5, Triadan 11, CS). Another Triadan 11 showed 3 completely separated pulp compartments with pulp horn 5 having a separate root canal within the distal root.

### Physical contact between roots of adjacent teeth.

**Fig. 8:**
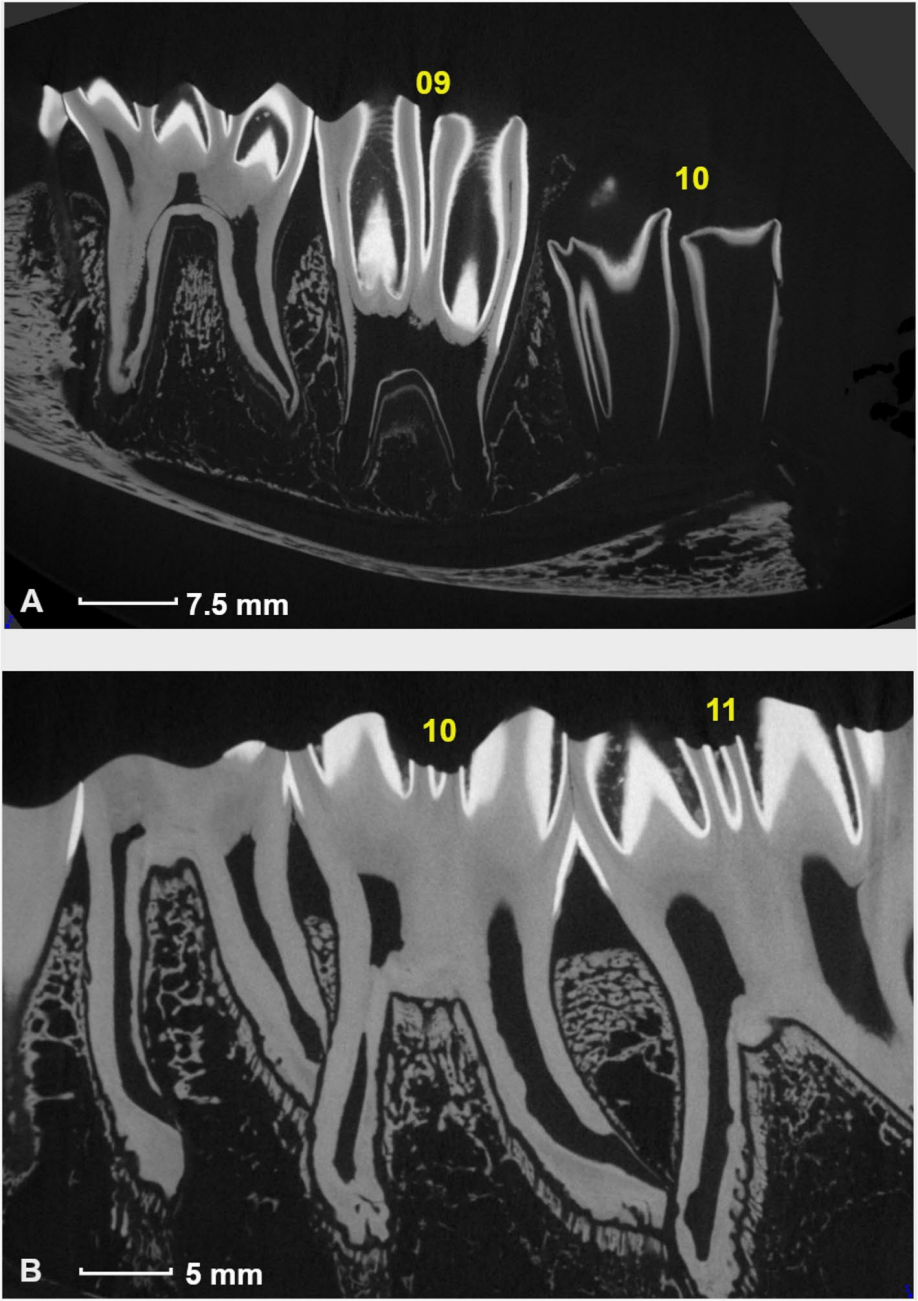
Contact and morphological adaptations of the roots of adjacent mandibular cheek teeth. A. Triadan 09 and 10 showing no contact in a 2 years and 2 months old alpaca. Note the start of the divergence of the developing roots of Triadan 09 in this relatively young tooth. Triadan 10 is only at the start of its development lacking a clear structure of the roots. B. Obvious contact between the distal root of Triadan 09 and the mesial root of Triadan 10 in a 7 years and 8 months old alpaca. Note the flattened distal root of Triadan 09. The distal root of Triadan 10 also contacts the mesial root of Triadan 11 resulting in comparable morphological changes to the root tip

With increasing dental age, the roots of mandibular molars were observed to become longer and deviate in a mesial or distal direction, resulting in physical contact between roots of adjacent teeth (Fig. 8).

These contacts were infrequently found between adjacent deciduous teeth (07–08: 17%). Most often they were recorded between Triadan 09–10s (80%; 8/10) and 10–11s (67%; 6/9). Root tips of deciduous 08s and permanent 09s, or permanent 08s and 09s contacted in 50% of examined cases each (3/6 and 4/8 respectively). In all animals older than 3 years, the tip of the distal root of mandibular Triadan 09s contacted the mesial root of Triadan 10s. Close contacts between adjacent roots led to a varying degree of morphological adaptation of involved roots in the majority of cases.

### Sub-occlusal dentinal thickness

**Fig. 9:**
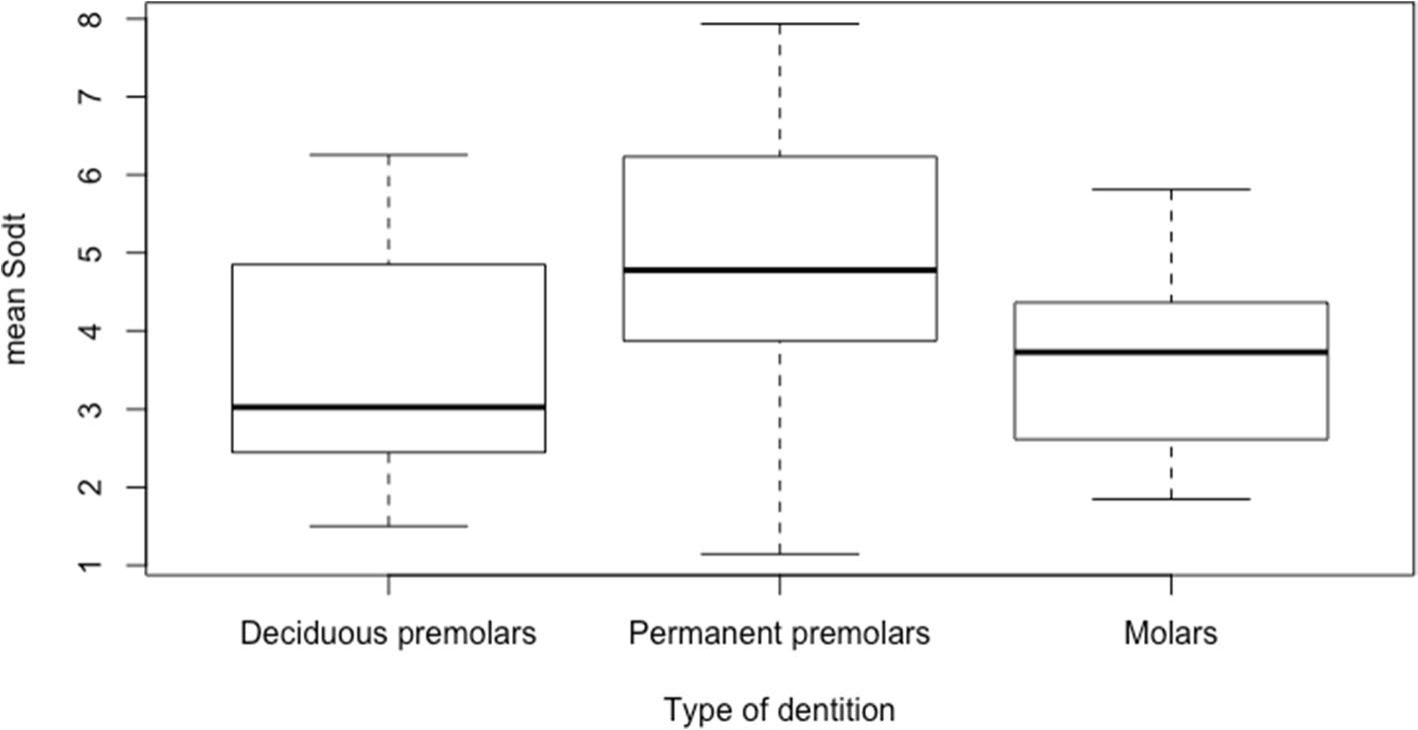
Boxplots of the mean sub-occlusal dentinal thickness for deciduous premolar, permanent premolar and molar teeth. Permanent premolars show a significantly higher mean SODT in comparison to the other types of dentition

The SODT was measured in 12, 8 and 27 deciduous premolar, permanent premolar and molar cheek teeth resulting in 47, 16 and 114 measurements, respectively. Overall, SODT ranged from 1.11 mm measured over pulp horns 2 and 4 in a deciduous Triadan 08 (dental age 2 years and 2 months) to 8.12 mm covering pulp horn 1 in a permanent Triadan 08 (dental age 2 years and 4 months). The mean SODT measured in deciduous premolar, permanent premolar and molar cheek teeth ranged from 1.50 to 6.26 mm (mean of 3.51 ± 1.45 mm), 1.15 to 7.94 mm (mean of 4.90 ± 2.24 mm) and 1.85 to 5.81 mm (mean of 3.56 ± 1.08 mm), respectively. A statistically significant higher mean SODT was found for permanent premolar cheek teeth in comparison to deciduous premolar (*P* = 0.002) and molar cheek teeth (*P* = 0.002) (Fig. 9).

Focusing specifically on molars, no statistically significant difference in mean SODT between female (3.39 ± 1.17 mm), male (4.20 ± 0.49 mm) and male castrated animals (3.34 ± 1.18 mm) was found. Additionally, mean SODT did not differ significantly for molars in the right (3.65 ± 1.07 mm) versus left (3.31 ± 1.16 mm) mandibular arcades, nor between Triadan positions ‘09’ (3.65 ± 1.08 mm), ‘10’ (3.39 ± 1.21 mm) and ‘11’ (3.63 ± 1.06 mm). Nevertheless, dental age was strongly associated with the mean SODT in the studied molars (*P* < 0.001). Older molars showed lower mean SODT values in comparison to younger specimens (Fig. 6). With every increase in dental age with 1 year, the mean SODT at the level of a specific mandibular molar cheek tooth is expected to decrease with 0.21 mm (95% CI 0.13–0.28 mm).

At pulp horn level within the same molar, SODT values did not differ significantly between the different pulp horns ((PH1 (3.32 ± 1.17 mm), PH2 (3.64 ± 1.53 mm), PH3 (3.77 ± 1.25 mm), PH4 (3.67 ± 1.23 mm) and PH5 (3.43 ± 1.83 mm)). Again, dental age was the only factor significantly associated with the SODT covering a specific pulp horn at the level of the mandibular molars (*P* < 0.001).

## Discussion

The objective of the present study was to improve understanding of the (pulpal) anatomy of mandibular cheek teeth which is a prerequisite to elaborate modern treatment options for apical disease in this species, including tooth sectioning followed by partial extraction, and endodontic procedures. Furthermore, it was of particular interest to investigate the risk of causing iatrogenic pulp exposure when floating cheek teeth in alpacas. Therefore μ-CT studies were performed on mandibular cheek teeth of varying dental age. Detailed three-dimensional reconstructions and two-dimensional images with a spatial resolution of approximately 0.05 mm were acquired for visualization and specific measurements.

Alpacas are currently classified within the order Artiodactyla, suborder Tylopoda [19]. Despite belonging to a different suborder, cheek teeth in alpacas show great similarities with cheek teeth in ‘true ruminants’. Our results confirm the presence of a selenodont occlusal surface in alpaca cheek teeth which shows sharp ridges and points specifically accustomed to the improvement of grinding efficiency [20]. This irregular occlusal surface develops due to varying wear characteristics of the different distinguishable calcified dental tissues [21]. The natural diet of alpacas in South America consists of native grasses and forbs, where they are never fed concentrates, supplements and are rarely fed cured hay [22]. Ingested forages are generally high in fiber and low in energy. When combined with yearly cycles of “feast and famine” in the Peruvian Altiplano, nutritional challenges in their natural habitat arise, necessitating the development of a highly efficient digestion. Feeding outside of South America is generally richer and doesn’t include a lean time, thereby predisposing the animals to become obese. Furthermore, the use of different feedstuffs and differing management conditions might predispose alpacas to the development of specific dental disorders, as is also perceived in domesticated horses [5, 23].

Distinct differences in general anatomy between deciduous and permanent Triadan 08s were observed. The deciduous Triadan 08 is a large rectangular tooth consisting of three columns each with an infundibulum. Its permanent successor is much smaller and only comprises of one column and infundibulum. These differing characteristics can easily lead to confusion in the identification of specific mandibular cheek teeth in the less experienced practitioner. Up until now, contradictory statements can be found concerning the presence of a specific infundibulum at the level of permanent Triadan 08s [3, 11]. Our results confirm the presence of one infundibulum at the level of this tooth. In newly erupting Triadan 08s, only an enamel infolding at the caudal border of the tooth is visible. In time, the infundibulum can disappear after years of intensive attrition and abrasion. The youngest recorded permanent Triadan 08 lacking an infundibulum in this study was 4 years and 1 month of dental age.

The presence of a previously described ‘llama buttress’ in llama and alpaca teeth was confirmed in studied alpaca mandibular molar teeth. This dental characteristic may be helpful in age estimation of New World Camelids. Age can be estimated relatively easy based on tooth eruption times in younger animals up to 3 years of age. However, in older animals, age estimation remains difficult and has been proposed to be based on wear patterns [15]. With increasing age, the llama buttress gradually disappears due to constant attrition, abrasion and eruption thus restricting its presence only in erupting teeth. However, further research into the value of this ‘llama buttress’ for age estimation is necessary as multiple factors including feeding management practices may have an influence on its presence.

The pulp system in mandibular cheek teeth shows some well-documented age-related variations in various other species, including humans and equids [8, 24]. Historically, a lack of communication between the pulp tissue in the mesial and distal column of alpaca cheek teeth was generally accepted [2, 3, 12]. However, a more recent study revealed the existence of a CPC in newly erupted cheek teeth [11]. Over time, the development of distinct roots and the internal continuous deposition of secondary dentin leads to narrowing and eventual segmentation of the pulp chamber as has been demonstrated before in other hypsodont species [8, 11]. The present study provides updated detailed insights into the timeframe where pulpal segmentation occurs with variations related to specific Triadan positions. The earliest sign of pulpal segmentation was recorded in a deciduous 08 with a dental age of 2 years. However, a CPC could be found in a permanent Triadan 08 with a dental age of 6 years and 10 months, illustrating aforementioned variation. Whether the pulp has undergone segmentation is of importance when dealing with apical disease. The authors have diagnosed pulpitis and apical disease in separated pulp segments, leaving other segments in the tooth healthy. This enables the option to only extract the diseased part of the tooth following tooth sectioning [3]. When performing this technique in a relatively young tooth with a pulpal connection between both segments, pulpitis can be iatrogenically induced if exposed pulp tissue is not sealed off. The expected dental age for pulp chamber segmentation in the deciduous mandibular premolars is around 2 years. Based on our results, a wider age variation is seen for permanent Triadan 08s (dental age range 6.5–11.5 years). Furthermore, pulpal segmentation can be expected between 1.5 and 2.5, between 2 and 5 and between 3 and 3.5 years of dental age in Triadan 09s, 10s and 11s respectively. However, individual variation should always be accounted for. If tooth sectioning is performed in specific cases, thorough inspection of the remaining sectioned tooth surface should be carried out to exclude macroscopically visible connections to the pulp system. If these are detected, a vital pulpectomy of the remaining segment should be performed. Presurgical CT-scan analysis of the patient’s skull not only helps in identifying diseased teeth but also substantially contributes to the characterisation of the individual tooth’s pulp system. However, standard CT-scan analysis of several alpaca skulls was unable to detect communications between pulp horns within the same column, whereas our study results clearly identified communications in all examined teeth [11]. These contradictory findings can be attributed to resolution differences between standard CT and μ-CT acquired images, the latter enabling visualization of the smallest interpulpal communications that are more easily missed in standard, clinical settings.

Our results and 3-D renderings of the pulp tissue conformations in different cheek teeth can provide a solid basis for the development of endodontic procedures in this species. Further research is warranted to optimize specific protocols for alpacas. The authors believe a staged procedure can be of particular value in this species. Important restrictions remain the limited access to the oral cavity and possible economical limitations in this species. Case selection is of uttermost importance as the apical region and periodontal space are often severely compromised, rendering endodontic treatments ineffective. Therefore, preventative and regular examinations are necessary to allow the early diagnosis of dental pathology. The importance of tooth saving techniques in alpacas should not be underestimated given the limited number of cheek teeth contributing to the occlusal surface.

Our study showed that with increasing age, roots of mandibular cheek teeth were characterized by varying degrees of deviation. Diverging of roots, more frequently observed in older cheek teeth, might complicate straight-forward oral extraction, even necessitating sectioning the tooth midway with subsequent extraction of both halves. Root divergence can be evaluated based on standard radiographic projections of the mandibular apical region as part of the diagnostic work-up of animals with apical disease [11]. Sectioning of multi-rooted teeth to create multiple single-rooted segments can be performed as is routinely done in canine or feline dentistry [25–27]. This technique is contraindicated in younger animals given the more vertical direction of their roots and the fact that the sectioned and therefore weakened tooth halves are more prone to fracturing secondary to forces exerted by the extraction forceps as they consist of a relatively thin outer wall and a larger internal pulp canal system. This complication is also described in cats were single-rooted crown-root segments created through sectioning are more likely to fracture during leverage [27–29]. Additionally, the distance between the occlusal surface and the bifurcation of the roots is rather large in alpacas which makes sectioning technically more demanding. The authors perform sectioning by utilizing a dental unit with a high-speed hand piece and a fissure bur or lindeman bur type. Access to more caudally affected cheek teeth is limited due to the relative rostral location of the lip commisures. To avoid unintentional damage to healthy oral tissues, procedures are advised to be performed under visual guidance with a dental oroscope. After finishing tooth sectioning, the authors prefer to extract the created segments orally using a combination of a small animal right angled extraction forceps, and/or a custom made extraction forceps when additional working length is required. Only in exceptional cases in which oral extraction proves to be unsuccessful, a lateral transcortical approach is needed to extract more distally located cheek teeth. In specific cases with extensive abscessation, a lateral transcortical approach can also be beneficial to allow for sufficient post-operative drainage by fixing a drain at the level of the extraction site.

The disto-mesial contact between roots of adjacent teeth with subsequent deviation, deformation, shortening or partial destruction of the apical ends of the specific roots has been reported previously [11]. The present μ-CT study could not find evidence to validate these findings. However, the presence of disto-mesial contacts was confirmed and recorded most frequently between Triadan 09 and 10. Some degree of morphological adaption at the apical aspect was observed in the majority of these roots. Nevertheless, based on our findings these could not be categorized as destructive lesions. It may be hypothesised that these morphological adaptations could be responsible for varying inflammatory changes in the apical region of a root, potentially increasing the risk of anachoretic infection. Furthermore, these root contacts may also play a facilitating role in the dissemination of apical infection towards adjacent mandibular cheek teeth as is frequently observed in alpacas (personal observation). Root tip deformation due to these contacts may as well contribute to the development of apical disease in conjunction with often diagnosed deep periodontal disease. The limited height of interproximal alveolar bone further contributes to a rapid invasion of the apical region (Fig. 8B) [4]. Further histological research of these specific contact regions can be of value to draw more definite conclusions regarding the pathogenesis of apical disease in alpacas.

Secondary dentin production is a continuous, age-related and dynamic process that prevents occlusal pulp exposure secondary to occlusal wear [24, 30, 31]. Little is known regarding the factors that may influence this process in hypsodont species [31]. SODT wear and production are balanced throughout the life in the hypsodont dentition of other domesticated species providing strong motives to accept that the deposition of secondary dentin is at least partly regulated by stimulation at the level of the occlusal surface [32, 33]. The present study showed significantly higher values of mean SODT in permanent premolars compared to deciduous premolars and molars. This difference could be attributed to the relatively low rate of attrition in permanent premolars given their fairly short clinical crown in comparison to the adjacent molars represented by a height difference in the mandibular occlusal surface. Basal secondary dentin production thereby exceeds the natural wear rate in these specific teeth. Additionally, this hypothesis also provides an explanation for the seemingly remarkable increase of SODT with increasing age in studied permanent premolars. In contrast, a decrease in SODT in deciduous premolar and molar cheek teeth with increasing age can be expected. Unfortunately, specific statistical analyses at the level of the premolars, necessary for drawing clear conslusions, remain impossible given the low sample size of these specific teeth. Nevertheless, a similar trend has been previously observed in mandibular cheek teeth of horses [32]. With increasing age, a disruption of the balance of sub-occlusal dentin wear and production may occur. High occlusal wear in these teeth initiates the deposition of relatively large amounts of secondary dentin above the involved pulp horns. With increasing age, odontoblasts in older cheek teeth in alpacas may not be able to produce sufficient amounts of secondary dentin in response to a continuous mechanical stimulation anymore, leading to an overall decrease in mean SODT.

Current recommendations in alpaca dentistry state that floating of cheek teeth should not be performed on a regular basis as is done in horses [3]. Abnormalities of wear benefiting from specific reduction, which are routinely encountered in equid teeth, are less frequent findings on oral examinations in alpacas [4, 34–37]. Our study results emphasize the risk of iatrogenically opening pulp canals when floating the occlusal surface of mandibular cheek teeth in this species. The SODT in our study population was as low as 1.11 mm in some pulp horns. The SODT was lower than 2, 3, and 4 mm in 13.1, 38.1 and 61.4% of performed measurements, respectively. However, a lot of variability was seen as also previously reported in horses, where the smallest SODT in equine cheek teeth and incisors was reported to be 2 mm and 0.7 mm, respectively [32, 38]. In contrast to current practice in equine dentistry, dental floating is not routinely performed in alpacas. Therefore, the SODT measurements reported in the present study may be considered representative for the natural SODT in this species. Iatrogenic pulp exposure as a cause of apical infection has been demonstrated in equids following aggressive dental floating [39, 40]. The occurrence of severe abnormalities of wear in alpacas has been reported in a large prevalence study [4]. In these severe cases, reduction of dental overgrowths can be beneficial for improvement of oral comfort. However, single stage reduction of these overgrowths would likely lead to occlusal pulp exposure in a majority of cases as has been previously demonstrated in horses [41, 42]. Staged reductions are therefore recommended. Additionally, the heat generated through use of powered rotary tools can be a causative factor for inducing thermal pulp necrosis, which can be responsible for postponed occlusal pulp exposure only appearing several years after the performed dental treatment [39, 43]. Intermittent or continuous water cooling has been proven beneficial to reduce pulp temperature increase when using motorized dental equipment [43]. Additional research, focusing on teeth diagnosed with specific wear abnormalities can aid to draw more definite conclusions and formulate definite recommendations.

This study is the first to utilize μ-CT examinations to gain a more thorough understanding of mandibular cheek teeth anatomy in alpacas. The acquired detailed images provide novel insights supporting the development of advanced dental treatment strategies in this species. The cross-sectional study design is an important limitation of the present study. Due to dose limitations, μ-CT examinations cannot be performed on live animals unabling a longitudinal study design in specific teeth over time. Also, practical feasibility concerning optimal sample positioning in live animals is questionable. Despite this study limitation, sufficient teeth within different age intervals have been selected to provide insights in the normal development and evolution of the pulp system at specific Triadan positions. However, selection bias cannot be ruled out given samples were retrieved from deceased animals admitted to the pathology department. Given important structural differences between mandibular and maxillary cheek teeth, results obtained in the present study are expected to be non-extrapolatable. Additional research focusing on specific anatomy, root contacts and SODT at the level of maxillary cheek teeth appear to be necessary to draw definite conclusions.

## Conclusions

In conclusion, detailed knowledge regarding age-dependent mandibular cheek tooth anatomy in alpacas is provided. Current findings support the development of modern treatment strategies in this species and provide general guidelines for gross decision making concerning specific dental treatments. Ideally, tooth and pulp morphology of a specific tooth should be evaluated using medical imaging techniques as part of a diagnostic work-up, prior to performing advanced dental treatments. Morphological adaptations at the apical aspects of contacting roots can be confirmed. The disto-mesial contacts and the little quantities of occlusal alveolar bone between adjacent roots can possibly be facilitating factors in the development of apical infection. A cautious approach with regards to dental floating is warranted given the SODT over an individual pulp horn can be as low as 1.11 mm. Further research is warranted to study the SODT over cheek teeth diagnosed with specific wear abnormalities.

## Data Availability

The datasets generated and/or analysed during the current study are not publicly available due to ongoing research projects but are available from the corresponding author on reasonable request.

## Abbreviations

CPC: Common pulp chamber
CS: Columnar segmentation
CT: Computed tomography
SODT: Sub-occlusal dentinal thickness
μ-CT: Micro-computed tomography
